# Identifying malaria vector breeding habitats with remote sensing data and terrain-based landscape indices in Zambia

**DOI:** 10.1186/1476-072X-9-58

**Published:** 2010-11-05

**Authors:** Julie A Clennon, Aniset Kamanga, Mulenga Musapa, Clive Shiff, Gregory E Glass

**Affiliations:** 1 Department of Molecular Microbiology and Immunology, Bloomberg School of Public Health, Johns Hopkins University, Baltimore, MD USA; 2 Department of Biostatistics and Bioinformatics, Rollins School of Public Health, Emory University, Atlanta, GA USA; 3 The Malaria Institute at Macha, Macha Mission Hospital, Choma, Zambia

## Abstract

**Background:**

Malaria, caused by the parasite *Plasmodium falciparum*, is a significant source of morbidity and mortality in southern Zambia. In the Mapanza Chiefdom, where transmission is seasonal, *Anopheles arabiensis *is the dominant malaria vector. The ability to predict larval habitats can help focus control measures.

**Methods:**

A survey was conducted in March-April 2007, at the end of the rainy season, to identify and map locations of water pooling and the occurrence anopheline larval habitats; this was repeated in October 2007 at the end of the dry season and in March-April 2008 during the next rainy season. Logistic regression and generalized linear mixed modeling were applied to assess the predictive value of terrain-based landscape indices along with LandSat imagery to identify aquatic habitats and, especially, those with anopheline mosquito larvae.

**Results:**

Approximately two hundred aquatic habitat sites were identified with 69 percent positive for anopheline mosquitoes. Nine species of anopheline mosquitoes were identified, of which, 19% were *An. arabiensis*. Terrain-based landscape indices combined with LandSat predicted sites with water, sites with anopheline mosquitoes and sites specifically with *An. arabiensis*. These models were especially successful at ruling out potential locations, but had limited ability in predicting which anopheline species inhabited aquatic sites. Terrain indices derived from 90 meter Shuttle Radar Topography Mission (SRTM) digital elevation data (DEM) were better at predicting water drainage patterns and characterizing the landscape than those derived from 30 m Advanced Spaceborne Thermal Emission and Reflection Radiometer (ASTER) DEM.

**Conclusions:**

The low number of aquatic habitats available and the ability to locate the limited number of aquatic habitat locations for surveillance, especially those containing anopheline larvae, suggest that larval control maybe a cost-effective control measure in the fight against malaria in Zambia and other regions with seasonal transmission. This work shows that, in areas of seasonal malaria transmission, incorporating terrain-based landscape models to the planning stages of vector control allows for the exclusion of significant portions of landscape that would be unsuitable for water to accumulate and for mosquito larvae occupation. With increasing free availability of satellite imagery such as SRTM and LandSat, the development of satellite imagery-based prediction models is becoming more accessible to vector management coordinators.

## Background

Malaria transmission throughout Africa is heterogeneous in space and time [1,2]. In the continent, it is estimated that 609 million people are at risk for malaria [3]. In Zambia, 34% of the population live in endemic risk areas while 48% of the population are in epidemic risk [4,5]. In epidemic areas malaria control programmes could make significant inroads in morbidity and mortality [3]. Recent analyses [6,7] suggest that larval control could play an important role in future control programmes, especially under these circumstances. In southern Zambia, malaria transmission is mainly associated with *Anopheles arabiensis *[8,9]. The prevailing climatic conditions in this dry, sub-humid environment restrict *Anopheles gambiae *complex and other anopheline mosquitoes, and limit the availability of suitable breeding habitats for *An. arabiensis*. Consequently, because of the restricted breeding habitats, malaria transmission risk is expected to be tightly clustered in space and time. Thus, identifying anopheline breeding habitats would allow focused control interventions to interrupt malaria transmission.

However, the identification of larval breeding sites is challenging. In southern Zambia, typical habitats for *Anopheles *larvae are partly sunlit, pools ranging in size from foot prints, to ponds, to slow moving streams [10]. *Anopheles *breeding habitats develop during the rainy season after the heavy rains, but begin to disappear at the start of the dry season until few or none remain. Such conditions will generate a complex dynamic of colonization, extinction and re-colonization of local anopheline populations, and help drive the seasonality of malaria transmission. Permanent breeding habitats that may act as "sources" of re-infestation in the wet season could represent targets for mosquito control interventions [11-13]. However, ground-based monitoring of potential aquatic breeding sites is labour-intensive, expensive and too difficult to maintain, except when these targets are few in number, easily accessible and well-defined [14].

A practical alternative approach is needed to define and identify small, broadly distributed larval breeding sites over broad geographic regions. One approach is statistical analysis with remotely-sensed characterizations of the environment and derived data products to identify and locate suitable breeding sites. Topographic data and derived indices are widely used by ecologists to describe landscape terrain and predict plant and animal species distributions. Raw elevation and slope are the most commonly used, but, increasingly, topographic indices (e.g., topographic wetness) and classifications (e.g., landform) based on topographic data are being evaluated [15]. Additionally, hydrological models are being applied to determine water drainage patterns. Following the trend in ecological studies, studies of vector populations have begun exploring the relationships of topographic descriptors with the distributions of mosquitoes. Some studies have modelled hydrology to examine the impact of distance to streams on disease risk [16], while others have examined the influence of water flow on the spatio-temporal dynamics on mosquito populations [17-19].

The objective of this study was to describe the spatial distribution of habitats of potential anopheline larval habitats in southern Zambia, by performing larval water surveys at the end of one rainy season, prior to the onset of the following season and at the end of a second rainy season. Different sources of readily available elevation data and terrain-based methods were evaluated to assess modelling approaches and data sources for predicting the abundance and distribution of such habitats. The long-term task is to develop methods for sustainable control of *Anopheles *larval populations that are targeted in space and time.

## Methods

### Study area

**Figure 1:**
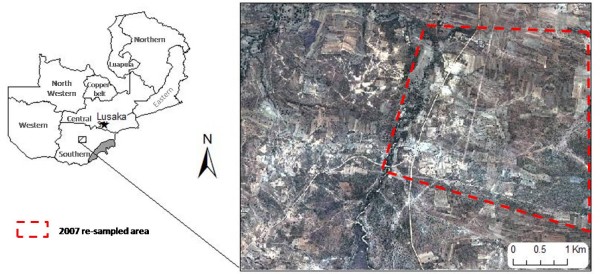
**The Nachiko Study Area located in Southern Province, Zambia shown with QuickBird imagery**.

Located 40 km south of the Kafue Flats in Zambia's Southern Province, the study area was centered on the Nachiko seasonal stream at 26° 52' 52" E, 16° 20'51" S (Figure 1 - map of study area). There are three seasons, rainy (November - April), cool-dry (May - August) and hot-dry (August - November) with the majority of the 60-100 cm average annual rainfall occurring in the rainy season. During the rainy season, water accumulates and remains flowing until the last weeks when water pools develop. The Nachiko Stream is a tributary to the perennial Munyeke River on the northern border of the study area. Various ponds, seasonal water pools and natural springs occur in the area. The landscape is predominately covered by munga scrub and grasslands, interrupted by tree cover over rivers and streams. In addition, there are agricultural fields of maize and peanuts. Approximately 240 households are in the area. The residential compounds consist of sleeping houses primarily constructed of brick, and peri-domestic structures included cooking shelters, maize storage, cattle corrals, goat and pig pens, chicken coups and pigeon houses. *Plasmodium falciparum *malaria transmission occurs seasonally with peak transmission in March and April corresponding with increased numbers of *An. arabiensis *and *Anopheles funestus *mosquitoes [9]. However *An. funestus *was locally extirpated following a drought in 2004-2005 [20]. Few residents had bed nets in 2006, and coverage was sporadic in 2007. In contrast, after the implementation of Zambia's national bednet campaign in October-November 2008, bednet coverage became evenly dispersed through the communities.

### Study design

A ground survey of ~18 km^2 ^was conducted on the ground by a field crew to search for water pools and aquatic habitats suitable for anopheline mosquitoes between March 19, 2007 and April 5, 2007. Global positioning system receivers (GPS) were used to conduct an extensive survey of the region, to collect ground control points and identify the location of water pools. In addition, residents were asked the locations of sites where they gathered water. Because most transient water pools disappeared after a day or two following a heavy rain leaving a subset of water pools more suitable for mosquito breeding, sampling for larvae was done during two or more days after a rain. Locations of water sites were collected using a Trimble XM GPS applying one minute of location averaging with a precision dilution of point of ≤4. Surveys were repeated on October 17 - 20, 2007 prior to the onset of rainy season to identify sites that still retained water and larval anophelines. A third survey was conducted at the end of the following rainy season from March 26 - April 4, 2008 to determine if breeding sites persisted from season to season. The October 2007 survey included the entire original study area, while the follow up March - April survey encompassed the area from the central stream and east.

### Digital elevation data

Two digital elevation models (DEM) for the area with 1 m horizontal resolution, were evaluated; the Shuttle Radar Topography Mission (SRTM) version 3 DEM http://dds.cr.usgs.gov/srtm/version2_1/SRTM3/ with 90 m pixels and Advanced Spaceborne Thermal Emission and Reflection Radiometer (ASTER) (U.S. Geological Survey) DEM with 30 m pixels. Elevation values in both DEMs corresponded to the reflective surface on the Earth that could include soil surface, vegetation (including tree tops) or man-made structures.

SRTM imagery was collected during a 2001 space shuttle mission using a multi-frequency, multi-polarization radar system. Each pixel represented a 30 m average of elevation around each pixel's centre. The relative horizontal accuracy is ±15 m (90% circular error) with a relative vertical accuracy of ±6 m (90% vertical error). ASTER imagery was collected on June 28, 2007, and is derived from the near infrared bands 3N and 3B collected in 15 m optical stereo. It has a relative accuracy ≥10 m.

Nadir-viewing and backward-viewing bands of Level-1A imagery (15 m horizontal resolution) collected by the visible near infrared (VNIR) sensor on the ASTER satellite was used to create DEMs with a 30 m horizontal resolution. The DEM generation was performed using an automated stereo-correlation method with ephemeris and attitude data from the ASTER instrument and the Terra spacecraft platform instead of ground control points (NASA, https://lpdaac.usgs.gov/). The RMSE-xyz (root-mean squared) is generally more than 25 meters accurate.

### Topographic wetness

The digital elevation models were processed in Imagine 9.1 and imported into ESRI ArcGIS 9.1 (Redlands, CA). All imagery and point locations were geo-referenced to UTM zone 35S, WGS 1984. The ArcGIS extension Terrain Analysis Using Digital Elevation Models extension (TauDem; Tarboton, Utah State University, 2005, http://hydrology.neng.usu.edu/taudem/) was used to model water flow throughout landscape and subsequently calculate an inverse topographic wetness index (TWI).

The data were smoothed to fill in isolated elevation pits (or spikes) which typically represent errors or areas of internal drainage that interrupt the estimate of water flow. Slope and flow directions, were determined from the multiple direction algorithm (MDA) method [21], in combination with the flat area flow direction method [22]. The MDA method used the steepest slope of triangular facets allowing water to flow in any direction. Topographic wetness index is an indicator of potential moisture, assuming there is surface homogeneity of soil and vegetation. It is calculated using the ratio of upslope contributing area (*A*) and local slope (the tangent of slope).

### Topographic position index

Topographic position classifies the landscape by slope position (low, middle, high) and landform type (plain, valley, ridge) [23]. It was generated using ArcView 3.3 with an extension by Jenness (2006) [24]. The topographic position index (TPI) is the difference between the elevation at a point and the mean elevation of neighborhood cells. TPI values near zero are typical of flat or mid-slope locations. High values signify high areas, such as hill tops and ridges, while low values are indicative of valley floors. Because TPI is a scale dependent variable, a local and an area-wide scale were considered (500 m and 2 km). The 500 m neighbourhood helps in detecting local valleys and hills, while the 2 km neighbourhood enables identification of larger scale features such a large U-shaped valleys, gently sloped hills, and tops of plateaus. Information from the magnitude of the TPI and the area's slope, was used to classify the slope position (SP) according to Weiss (2001)[23]. This classification uses the TPI score standard deviations (SD) and slope values. Slope position classes created were valley, lower slope, flat slope, middle slope, upper slope and ridge (Table 1).

**Table 1:** Slope position classes defined by Weiss, 2001.

Slope Position Class	TPI	Slope
valley	<-1 SD	

lower slope	≥-1 SD and <-0.5 SD	

flat slope	≥-0.5 SD and ≤0.5 SD	≤5°

middle slope	>-0.5 SD and <0.5 SD	>5°

upper slope	>0.5 SD and ≤1 SD	

ridge	>1 SD	

SD = standard deviation, (>) = above mean, (<) = below mean

Ten landform (LF) classes (deep streams, shallow valleys and mid-slope drainage pathways, upland drainage areas, U-shaped valleys, plains, open slopes, upper slopes and mesas, local ridges and hills in large valleys, mid-slope of ridges and small hills in plains, high ridges) were generated by comparing standardized TPI values (standardized TPI = [TPI - TPI mean]/[TPI standard deviation]) for TPI values at 500 m (TPI_500_) and TPI values at 2 km (TPI_2000_) and slope using criteria developed by Weiss (2001) (Table 2).

**Table 2:** Landform classes defined by topographic indices and slope (Weiss 2001).

Landform Class	**TPI**_**500**_	**TPI**_**2000**_	Slope
deep streams	≤-1	≤-1	

shallow valleys and mid-slope drainage pathways	≤-1	>-1 and <1	

upland drainage are	≤-1	≥1	

U-shaped valleys	>-1 and <1	≤-1	

plains	>-1 and <1	>-1 and <1	≤5°

open slopes	>-1 and <1	>-1 and <1	>5°

upper slopes and mesas	>-1 and <1	≥1	

local ridges and hills in large valleys	≥1	≤-1	

mid-slope of ridges and small hills in plains	≥1	>-1 and <1	

high ridges	≥1	≥1	

### Landscape characterization

Band digital numbers (DN) and vegetation indices derived LandSat imagery were used to account for the influences of vegetation and soil moisture on potential locations of water pooling. The LandSat TM 5 (LS) imagery scene (courtesy of the Council for Scientific and Industrial Research [CSIR] - Satellite Applications Centre, South Africa) (30 m resolution) was acquired on April 17, 2007.

LandSat satellite imagery is composed of 7 bands. Each band measures an unique wavelength: Band 1 (0.45 - 0.52 μm), Band 2 (0.52 - 0.60 μm), Band 3 (0.63 - 0.69 μm), Band 4 (0.76 - 0.90 μm), Band 5 (1.55 - 1.75 μm), Band 6 (10.40 - 12.50 μm), Band 7 (2.08 - 2.35 μm). In order to discriminate potentially variations in vegetation, moisture and soil, LS band indices were considered for incorporation into analyses. LandSat band ratios are known to detect different soil, vegetation properties (e.g., iron oxide = [band 3/band 1], clay = [band 5/band 7] and vegetation = [band 2/band 4]) ([25,26]).

### Statistical analyses

Statistical tests were performed using Stata 8.2 (StataCorp, College Station, Texas). Univariate logistic regression was used to assess the association of terrain variables individually. Backwards general linear logistic regression (GLM) was applied using to create risk models for presence/absence (1/0) of water pools, *Anopheles *larvae and *An. arabiensis *larvae. Independent explanatory variables were selected based on the criteria of Pearson correlation coefficient <0.8. Absence of water was represented by 100 randomly chosen ground control locations. Explanatory variables which were assessed included terrain variables (slope, aspect, TWI, TPI_500 _and TPI_2000_) and LS variables (2:4, 5:7, and 3:1). To reduce the number of variables included in prediction modelling, TPI_500 _and TPI_2000 _were included in predictive models without SP or LF. Post-estimation specificity, sensitivity and receiver operating characteristic (ROC) scores were calculated for GLM models. Residuals of models were tested for spatial autocorrelation using the global spatial statistic, Moran's I with inverse distance weighting (ArcGIS version 9.3.1, Environmental System Research Institute, Redlands, CA). Moran's I tests if the observed spatial pattern of point values over the entire study area are random, clustered, or uniformly dispersed. Models were further assessed using a generalized linear mixed model (GLMM) approach with a logit link [27] in the R-software package 'glmmML' http://cran.r-project.org/web/packages/glmmML with the variable cluster (i.e., agglomerations of sites within 60 m of each other) as a random effect. Such models that include a random intercept allow accounting for the amount of unexplained variance that can arise due to the spatial proximity of sites (i.e., spatial dependence between near observations). Residuals derived from GLMM models were, also, tested for global spatial autocorrelation using global Moran's I.

## Results

### Mosquito species

**Figure 2:**
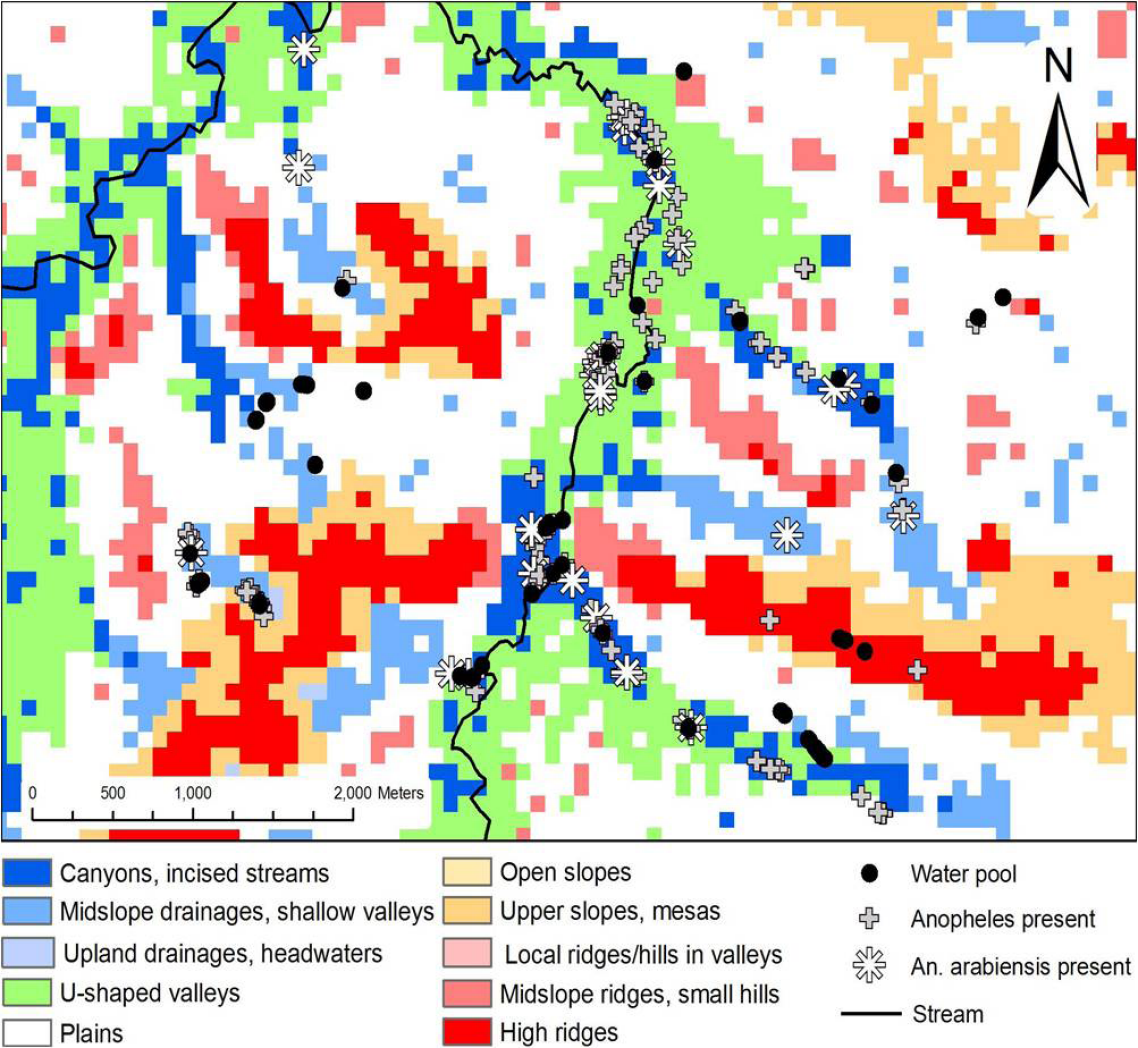
**Locations of water sites with anopheline larvae overlaid on landform types derived from SRTM Imagery**.

Two water-larval surveys were conducted. Of the 200 sites with water found during the first survey (Figure 2), 69% [n = 139] contained anopheline mosquito larvae. Of the anopheline sites, 26 [19%] contained recognized malaria vector species. Overall, nine anopheline species were identified. The maximum diversity at any site was 5 species. *Anopheles rufipes *was the most common mosquito found followed by *Anopheles squamosus, Anopheles coustani*, and *An. arabiensis *(Table 3). *Anopheles arabiensis *and *Anopheles quadriannulatus *were the only *An. gambiae *complex mosquito species identified in the study area. *Anopheles arabiensis *was found at 13 percent of all water sites (n = 26).

**Table 3:** Anopheline mosquito species identified.

	Total (N)	% of anopheline Positive sites	% of Total Sites
***An. arabiensis***	26	23	13

***An. quadriannulatus***	7	6	3.5

***An. rufipes***	51	46	25

***An. pretoriensis***	16	14	8

***An. leesoni***	1	1	<1

***An. rivulorum***	3	3	1

***An. longipalpis***	2	2	1

***An. coustani***	46	41	23

***An. squamosus***	50	45	25

During the October survey prior to the onset of the rainy season, only five water sites that were identified during the first survey still contained water, and none had any *Anopheles spp*. larvae. No additional water sites were found during this survey. During the March 2008 survey, 122 water sites were located along and to the east of the stream, and 51 contained *Anopheles *larvae of which five were *An. arabiensis *larvae. All of these 122 sites had been detected in 2007 and few new sites were identified far from the previous year's locations.

### Topographic position indices

Individually, TPI and SP terrain indices were correlated with the presence of water (P < 0.01) and anopheline mosquitoes (P ≤ 0.02). In general, terrain indices derived from SRTM data were better predictors than those derived from ASTER data and only two ASTER terrain indices (SP_2000 _OR = 1.54, 95% CI = 1.29 - 1.84, P < 0.001; TPI_2000 _OR = 0.95, 95% CI = 0.93 - 0.98, P < 0.001) were associated with the presence of water. Only SRTM TPI_2000 _(OR = 0.9, 95% CI = 0.81 - 0.99, P < 0.04) and SRTM SP_2000 _(OR = 2.07, 95% CI = 1.13 - 3.8, P < 0.02) predicted *An. arabiensis*.

Using SRTM to characterize the landscape, the majority of the area was composed of plains (50%) (Table 4) (Figure 2). While water drainage and valleys made up 32% of the area, more than three quarters (79.5%) of water sites were found in these land forms (U-shaped valleys OR = 2.77, 95% CI = 1.5 - 5.08, P < 0.001; incised streams (OR = 5.79, 95% CI = 2.66 - 12.64, P < 0.001) (Table 4). Anopheline larvae were further restricted and predominately (89%) found in deep streams (OR = 3.59, 95% CI = 2.05 - 6.27, P < 0.001) and valleys (OR = 2.66, 95% CI = 1.58 - 4.48, P < 0.001) while *An. arabiensis *larvae were almost completely restricted (96% of sites) to these landforms (streams OR = 2.87, 95% CI = 1.26 - 6.52, P < 0.02) (Table 4).

**Table 4:** SRTM landform classes for the study area and study sites by larval and *An. arabiensis *presence.

Landform	SRTM	Water Sites	*Anophele spp*. Positive	*An. arabiensis *Positive
Deep streams	133 (6.9)	28 (14)	19 (13.7)	4 (15.4)

Shallow valleys and mid-slope drainage pathways	119 (6.2)	8 (4)	4 (2.9)	1 (3.8)

Upland drainage areas	2 (0.1)	0 (0)	0 (0)	0 (0)

U-shaped valleys	361 (18.8)	43 (21.5)	37 (26.6)	9 (34.6)

Plains	966 (50.2)	71 (35.5)	42 (30.2)	6 (23.1)

Open slopes	0 (0)	13 (6.5)	9 (6.5)	3 (11.5)

Upper slopes and mesas	108 (5.6)	11 (5.5)	8 (5.8)	0 (0)

Local ridges and hills in large valleys	0 (0)	20 (10)	18 (12.9)	3 (11.5)

Mid-slope of ridges and small hills in plains	83 (4.3)	0 (0)	0 (0)	0 (0)

High ridges	151 (7.9)	6 (3)	2 (1.4)	0 (0)

Total	1923 (100)	200 (100)	139 (100)	26 (100)

N = Count, (%) = percentage

**Figure 3:**
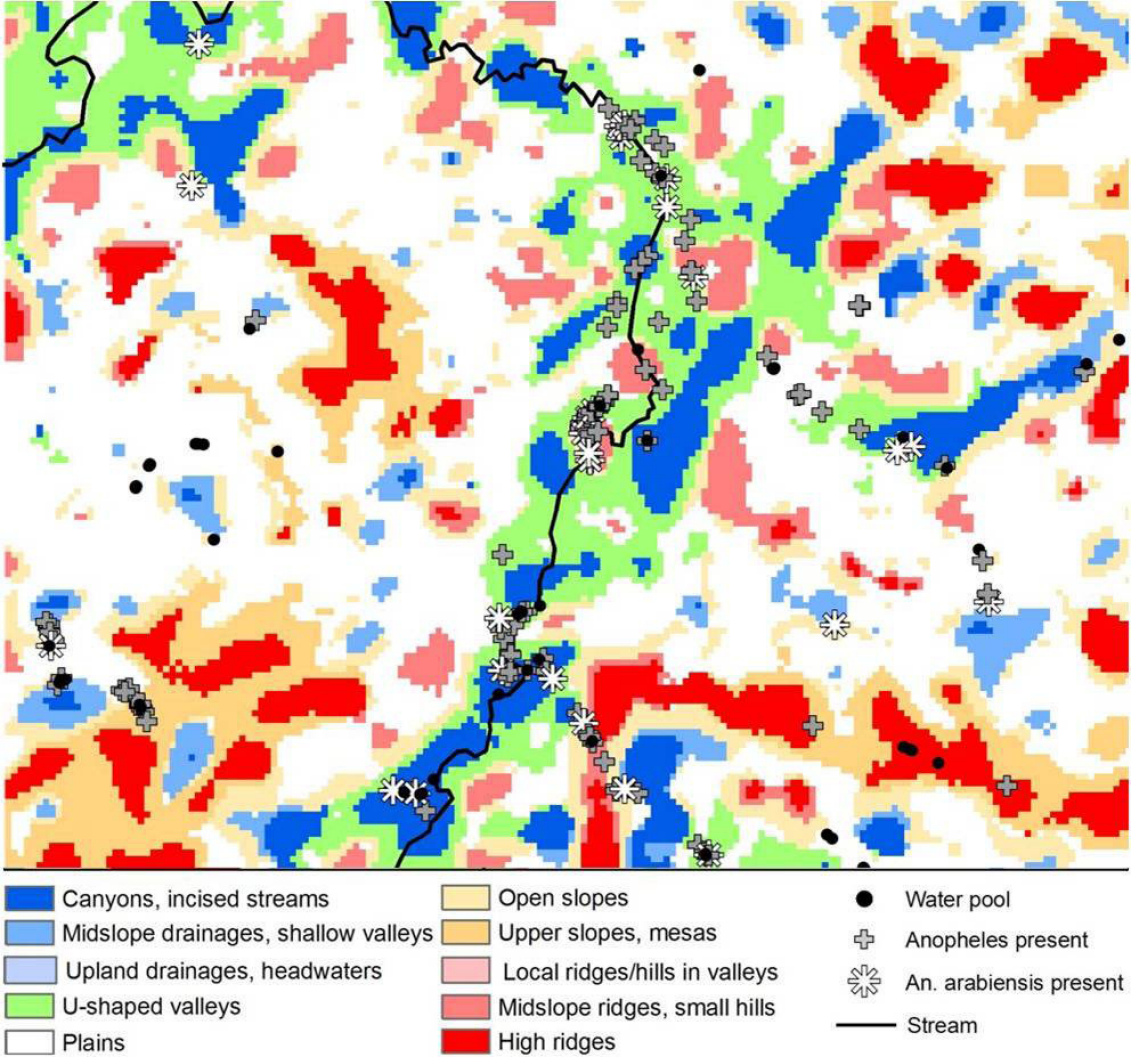
**Locations of water sites with anopheline larvae overlaid on landform types derived from ASTER Imagery**.

Landforms classified from ASTER provided a less defined characterization of larval habitats. For example, 43% of all larval sites and 54% of *An. arabiensis *sites were found in ASTER valleys and drainage pathways, which composed 22% of the area (Table 5) (Figure 3). No single ASTER classified landform type was associated water presence, but ridges predicted water absence (OR = 0.21, 95% = 0.08 - 0.56, P < 0.002). *Anopheles *presence was significant in valleys (OR = 2.18, 95% CI = 1.22 - 3.89, P < 0.001) and small hills in plains (OR = 2.85, 95% CI = 1.2 - 6.77, P < 0.02). None of the ASTER landform categories alone were significantly associated with presence of *An. arabiensis *larvae.

**Table 5:** ASTER landform classes at 500 m and 2,000 m for the study area and study sites by larval *Anopheles spp*. and *An. arabiensis *presence.

LandForm	ASTER	Water Sites	*Anopheles *Positive	*An. arabiensis *Positive
Deep streams	1733 (8.3)	28 (14)	19 (13.7)	4 (15.4)

Shallow valleys and mid-slope drainage pathways	952 (4.6)	8 (4)	4 (2.9)	1 (3.8)

Upland drainage areas	0 (0)	0 (0)	0 (0)	0 (0)

U-shaped valleys	1976 (9.5)	43 (21.5)	37 (26.6)	9 (34.6)

Plains	8167 (39.3)	71 (35.5)	42 (30.2)	6 (23.1)

Open slopes	2304 (11.1)	13 (6.5)	9 (6.5)	3 (11.5)

Upper slopes and mesas	2081 (10)	11 (5.5)	8 (5.8)	0 (0)

Local ridges and hills in large valleys	0 (0)	0 (0)	0 (0)	0 (0)

Mid-slope of ridges and small hills in plains	1396 (6.7)	20 (10)	18 (12.9)	3 (11.5)

High ridges	2168 (10.4)	6 (3)	2 (1.4)	0 (0)

Total	20777 (100)	200 (100)	139 (100)	26 (100)

Count (percentage)

### Multivariate Analyses

Further characterization of the remote sensing data and products was conducted to evaluate the presence of potential breeding sites (water presence) as well as those sites that yielded *Anopheles *spp as well as sites that yielded *An. arabiensis *larvae.

The best GLM logistic model (AIC = 306.6) found to predict the occurrence of water (ROC = 0.81) (Table 6) had four LS and SRTM landscape indices as the main predictors (Table 7). The sensitivity of this model was 92.0% and the specificity 49%. The variables from the LS-SRTM model were: TPI500, SRTM slope, SRTM aspect, and SRTM TWI. This model indicates that water was most likely to occur in local depressions (SRTM TPI500) with flattened slopes (SRTM slope). A classification of the LS-SRTM model with a cutoff probability of ≥0.5 resulted in 78.3% of sites being correctly classified. A model using ASTER data was 50 AIC units lower than the best model (Table 6) and presented a marginal predictive value (0.65) using three topographic variables (Table 7). The GLMM further increased model fit for the LS-SRTM model (AIC = 275.3) (Table 6), and had three topographic variables (TPI_500_, aspect and slope) as the main predictors (Table 8). The random intercept in the latter model was not significant (Intercept = -1.16, P-value = 0.12), suggesting a limited effect of proximity between sites in the prediction of water habitats. The GLMM, also, improved the model fit for the LS-ASTER model (AIC = 298.7), but its support was much less than the LS-SRTM model.

**Table 6:** Model comparisons for predicting the presence of water, *Anopheles *species larvae, and *An. arabiensis *larvae.

Presence	Contrast	GLM/GLMM	SRTM/ASTER	AIC	ΔAIC
Water	Random	GLM	SRTM	306.6	31.3

Water	Random	GLM	ASTER	366.2	90.9

Water	Random	GLMM	SRTM	275.3	**-**

Water	Random	GLMM	ASTER	298.7	23.4

					

*Anopheles*	Random	GLM	SRTM	238.4	13.4

*Anopheles*	Random	GLM	ASTER	297.4	72.4

*Anopheles*	Random	GLMM	SRTM	225	**-**

*Anopheles*	Random	GLMM	ASTER	252.1	27.1

					

*An. arabiensis*	Random	GLM	SRTM	82.2	**-**

*An. arabiensis*	Random	GLM	ASTER	110.8	28.6

*An. arabiensis*	Random	GLMM	SRTM	84.2	2

*An. arabiensis*	Random	GLMM	ASTER	112.1	29.9

					

*Anopheles spp*.	Water	GLM	SRTM	234.9	26.7

*Anopheles spp*.	Water	GLM	ASTER	241.9	33.7

*Anopheles spp*.	Water	GLMM	SRTM	208.2	**-**

*Anopheles spp*.	Water	GLMM	ASTER	213.7	5.5

					

*An. arabiensis*	Water	GLM	SRTM	151.9	**-**

*An. arabiensis*	Water	GLM	ASTER	153.4	1.5

*An. arabiensis*	Water	GLMM	SRTM	153.9	2

*An. arabiensis*	Water	GLMM	ASTER	155.4	3.5

**Table 7:** Predicting water presence compared to random locations using GLM

Model	Data Source	Variable	Odds Ratio	P > |z|	95% CI Lower	95% CI Upper
SRTM-LandSat	SRTM	slope	0.40	<0.001	0.27	0.61
	
	SRTM	TWI	1.29	0.02	1.04	1.59
	
	SRTM	aspect	1.003	0.012	1.00	1.005
	
	SRTM	TPI_500_	0.65	<0.001	0.55	0.75

						

LandSat -ASTER	ASTER	TPI_2000_	0.96	0.005	0.94	0.99
	
	LandSat	3:1	0.005	0.006	0.0001	0.22

**Table 8:** Predicting water presence compared to random locations using GLMM

Model	Data Source	Variable	Odds Ratio	P > |z|	95% CI Lower	95% CI Upper
SRTM-LandSat	SRTM	slope	0.38	0.01	0.18	0.81
	
	SRTM	aspect	1.01	0.02	1.00	1.01
	
	SRTM	TPI_500_	0.65	<0.001	0.52	0.82

						

LandSat -ASTER	ASTER	TPI_500_	1.29	0.01	1.06	1.57
	
	ASTER	slope	0.75	0.03	0.59	0.97
	
	ASTER	TPI_2000_	0.81	0.002	0.70	0.92

The model that best predicted (AIC = 238.4) (Table 6) *Anopheles spp *mosquito larval habitats had a ROC value of 0.85 and SRTM topographic indices and LS ratios (SRTM slope, SRTM TPI500, SRTM TPI20, and TWI) as the main predictors (Table 9). The model predicted anopheline larvae present in local depressions (SRTM TPI500) with flattened slopes (SRTM slope). The model (cutoff probability ≥0.5) correctly classified 70% of sites (sensitivity = 81.3%, specificity 52%). The GLMM improved model fit (AIC = 221.4) and had only TPI_2000 _as the main predictor (Table 10). As observed with the water GLMM model, the random effects term of the *Anopheles spp *LS-SRTM model (AIC = 225) was not statistically significant (Intercept = -1.59, P-value = 0.051). However, the ASTER-LS GLMM model (AIC = 252.1) for *Anopheles spp *was found to have a significant random intercept (Intercept = -1.88, P-value < 0.001).

**Table 9:** Predicting *Anopheles *presence compared to random using GLM

Model	Data Source	Variable	Odds Ratio	P > |z|	95% CI Lower	95% CI Upper
LandSat-SRTM	SRTM	slope	0.47	0.005	0.27	0.80
	
	SRTM	TPI_500_	0.58	<0.001	0.47	0.71
	
	SRTM	TWI	1.36	0.002	1.05	1.76
	
	SRTM	TPI_2000_	0.94	0.03	0.88	0.99

						

LandSat-ASTER	LandSat	3:1	2.40e - 06	<0.001	3.15e - 09	0.002
	
	ASTER	TPI_2000_	0.95	<0.001	0.92	0.97
	
	LandSat	2:4	316321.9	0.007	30.83	3.25e + 09

**Table 10:** Predicting *Anopheles *presence compared to random using GLMM

Model	Data Source	Variable	Odds Ratio	P > |z|	95% CI Lower	95% CI Upper
LandSat-SRTM	SRTM	slope	0.3	0.004	0.13	0.67
	
	SRTM	TPI_500_	0.46	<0.001	0.32	0.65
	
	SRTM	TWI	1.66	0.02	1.1	2.5

						

LandSat-ASTER	ASTER	TPI_500_	1.24	0.02	1.04	1.48
	
	ASTER	TPI_2000_	0.81	0.001	0.72	0.92

The best GLM model (AIC = 82.2) (Table 6) of *An. arabiensis *breeding habitats had a very high predicting value (ROC = 0.92) and had SRTM and LS band ratios (SRTM slope, LS band ratio 2:4, LS band ratio 5:7, SRTM TPI500) as the main predictors (Table 11). The model predicted *An. arabiensis *larvae to be present in local depressions (SRTM TPI_500_) with flatten slopes (SRTM slope). In addition, there was a strong relationship between increased greenness (LS band ratio 2:4) with *An. arabiensis *presence. The model (probability cutoff = 0.5) correctly classified 71% of sites (sensitivity = 65.1%, specificity = 93%). The implementation of a GLMM on the same data did not produce any significant improvement of the model (AIC = 82.2-84.2), although the GLMM model did have a significant random intercept (Intercept = -21.34, P-value = 0.01) (Table 12).

**Table 11:** Predicting *An. arabiensis *presence compared to random using GLM

Model	Data Source	Variable	Odds Ratio	P > |z|	95% CI Lower	95% CI Upper
LandSat-SRTM	SRTM	slope	0.08	<0.001	0.02	0.32
	
	SRTM	TPI_500_	0.56	<0.001	0.41	0.77
	
	LandSat	5:7	93.9	0.03	18.9	2.06e + 20
	
	LandSat	2:4	6.24e + 10	<0.001	0.41	0.77

						

LandSat-ASTER	ASTER	TPI_500_	1.24	0.005	1.07	1.43
	
	ASTER	TPI_2000_	0.83	0.001	0.74	0.92
	
	LandSat	5:7	66.00	0.004	3.89	1119.57
	
	LandSat	2:4	2.97e + 10	0.008	512.39	1.72e + 18

**Table 12:** Predicting *An. arabiensis *presence compared to random using GLMM

Model	Data Source	Variable	Odds Ratio	P > |z|	95% CI Lower	95% CI Upper
LandSat-SRTM	SRTM	slope	0.08	<0.001	0.02	0.32
	
	SRTM	TPI_500_	0.56	<0.001	0.41	0.77
	
	LandSat	5:7	93.9	0.02	2.37	3726.71
	
	LandSat	2:4	6.24e + 10	0.03	18.91	2.06e + 20

						

LandSat-ASTER	ASTER	TPI_500_	1.23	0.02	1.04	1.47
	
	ASTER	TPI_2000_	0.83	0.004	0.72	0.94
	
	LandSat	5:7	99.09	0.006	3.67	2672.71
	
	LandSat	2:4	4.37e + 11	0.01	576.29	3.32e + 20

Further analyses were performed to determine if breeding sites for *Anopheles *spp or specifically *An. arabiensis *differed from the remaining sites with water. The best *Anopheles spp *GLM model (AIC = 234.9)(Table 6) had LS-SRTM (LS band ratio 2:4, LS band ratio 3:1, SRTM slope, SRTM TPI_500_) as the main predictors and marginally (ROC = 0.71) distinguished sites with *Anopheles *spp larvae from all other water sites (Table 13). The implementation of a GLMM increased fit (AIC = 208.2), with the best model having terrain variables TWI and TPI_2000 _as the main predictors (Table 14). The model did have a significant intercept (Intercept = -0.44, P-value < 0.001).

**Table 13:** Predicting *Anopheles *presence compared to water using GLM

Model	Data Source	Variable	Odds Ratio	P > |z|	95% CI Lower	95% CI Upper
LandSat-SRTM	SRTM	slope	0.53	0.0014	0.32	0.88
	
	SRTM	TPI_500_	0.87	0.03	0.77	0.99
	
	LandSat	3:1	0.00004	0.025	5.98e - 09	0.28
	
	LandSat	2:4	100336.2	0.03	2.71	3.72e + 09

						

LandSat-ASTER	LandSat	3:1	2.50e - 06	0.002	6.35E - 10	0.01
	
	LandSat	2:4	127191.3	0.021	5.88	2.75e + 09

**Table 14:** Predicting *Anopheles *presence compared to water using GLMM

Model	Data Source	Variable	Odds Ratio	P > |z|	95% CI Lower	95% CI Upper
LandSat-SRTM	SRTM	TWI	0.81	<0.001	0.73	0.91
	
	SRTM	TPI_2000_	1.46	0.03	1.04	2.03

						

LandSat-ASTER	ASTER	TPI_2000_	0.93	0.02	0.87	0.99

The best model (AIC = 151.9) (Table 6) of *An. arabiensis *had LS-SRTM (SRTM slope)(Table 15) as the main predictor, distinguishing *An. arabiensis *from other water sites only marginally (ROC > 0.64). The sensitivity was 100%, while the specificity was 0%. The GLMM model using LS-SRTM detected an association with slope (Table 16) and *An. arabiensis *presence along with a significant spatial effect (Intercept = -0.90, P-value < 0.04).

**Table 15:** Predicting *An. arabiensis *presence compared to water using GLM

Model	Data Source	Variable	Odds Ratio	P > |z|	95% CI Lower	95% CI Upper
LandSat-SRTM	SRTM	slope	0.36	0.02	0.15	0.85

						

LandSat-ASTER	ASTER	TWI	0.94	0.03	0.89	0.99
	
	ASTER	TPI_2000_	1.66	0.02	1.09	2.51

**Table 16:** Predicting *An. arabiensis *presence compared to water using GLMM

Model	Data Source	Variable	Odds Ratio	P > |z|	95% CI Lower	95% CI Upper
LandSat-SRTM	SRTM	slope	0.36	0.02	0.15	0.85

						

LandSat-ASTER	ASTER	TWI	1.66	0.02	1.09	2.51
	
	ASTER	TPI_2000_	0.94	0.03	0.88	0.99

## Discussion

Malaria transmission risk depends on the presence of specific anopheline species, on the characteristics and productivity of their breeding sites, their location in relation to human settlements, and on the effective dispersal range of the mosquitoes. Such characteristics determine the context in which malaria transmission occurs, and the type and extent of control actions that can be performed. The ability to locate larval habitats and understand their distribution in space and time is an important component in planning and implementing effective and sustainable vector control strategies [6,28,29]. It also would substantially shrink the geographic extent that needs targeting in any control programme. However, identifying and monitoring these conditions on a meaningful spatial scale is daunting in many circumstances.

The present study shows that processed remotely sensed data combined with field calibrations allows prediction of potential vector breeding habitats in areas characterized by highly seasonal malaria transmission. Specifically, large portions of region can be excluded from interventions with a high degree of certainty making it possible to prioritize target regions for surveillance, monitoring and treatment. The models presented in this study show strong internal validation, but have yet to be externally validated to determine the strength of these results for other areas. While local spatial variation increased fit for many models, it often failed to significantly affect the predictive value of the models.

In sub-tropical Africa the amount and seasonality of rainfalls drastically affects the occurrence and productivity of mosquito breeding habitats and limits the distribution of certain anopheline species [2,30]. In the Nachiko study area, a single rainy season with the lower rainfall amounts (600 - 1,000 mm) coupled with the low winter temperatures permit *An. arabiensis *and *An. funestus *to survive while *An. gambiae *does not. Moreover, such conditions preclude establishing rice cultivations and irrigation canals, reducing the chances of anthropogenic, large and highly productive breeding habitats. Under such circumstances, the most common breeding habitats are seasonal water pools of various sizes, gently flowing waters, and springs.

Most of these water pools were prone to drying, and nearly all disappeared during the dry season. *An. arabiensis *was found in water pools of various sizes, depths, sun illumination, and turbidity towards the end of the rainy seasons but was absent at the end of the intervening dry season in the few pools of water that remained. The various physical conditions in which *An. arabiensis *larvae were found precluded attempts to more specifically predict its spatial distribution with the available environmental monitoring tools. However, nearly all its breeding sites were in landforms classified as deep streams and valleys- a relatively limited portion of the study region. The multivariate analyses comparing *An. arabiensis *sites with other water containing locations suggested some distinctions. However, these were not sufficient to suggest that targeting of water sites based on these characteristics would be satisfactory. These results may occur because this species is more generalist in choosing breeding habitats when water is limited. This contrasts to areas with two rainy seasons where *An. arabiensis *is found with higher mosquito densities and appears to prefer clean, clear pools [10]. However, for models described here, sensitivity was typically high so that they rarely failed to identify potential sites. Consequently, few if any of the sites were likely to be missed - a major consideration in any control programme.

In the Nachiko Area, no habitats contained larval *An. arabiensis *during the driest months of the year (October-November). This suggests that there may be repeated recolonizations of the region near the start of the rainy season. Three kilometers to the north is a seasonal river that develops into a series of mostly clear water pools during the dry season. Larval *An. rufipes *have been found even during the driest of months suggesting that *An. arabiensis *may persist there at an extremely low population density (although none were observed). Given that *Anopheles *mosquitoes are not occupying aquatic habitats outside of seasonal large rivers, a focus towards surveying them during the dry season and applying larvicides may be a cost effective approach in controlling malaria vector mosquitoes near the study area.

When coupled with field surveys for calibration, satellite imagery allows the measurement of factors and the strength of their association in limiting the distributions of anopheline species both on large and local scales characterizing environmental conditions on wider extents than those that can be done on the ground. These data can act as surrogates of vegetation and moisture [31]. Fine-scale predictive models were developed based on topographic and satellite (LandSat TM5) imagery indices, which identify areas likely to hold water following the rains and predict aquatic habitats where anopheline mosquitoes (and specifically *An. arabiensis*) can be present.

However, these analyses also demonstrate that these data products may differ in their utility. For example, both SRTM and ASTER produce DEM products that can be used in land form classification. However, the SRTM derived products appeared to provide a better interpretation. These analyses demonstrate that the use of finer resolution satellite imagery (*e.g.*, ASTER) is not always better than slightly more coarse imagery (*e.g.*, SRTM) to use for predictive modelling. Information derived from Landsat imagery also appeared more successful than ASTER data in classification of breeding sites.

Topographic conditions conducive for water pooling are local depression with flat slopes. These locations are generally places with sandy-loam to clay soils where water will accumulate but because of the flat slopes do not drain. When coupled with soil moisture and vegetation indices derived from LandSat satellite imagery, it is apparent that vegetation, soil typing and moisture levels in conjunction with terrain-based modelling can limit the survey area needed to find the vast majority of suitable aquatic habitats for anopheline larval development. Topographic indices were important variables in predicting the presence of water pools during the mosquito season in Southern Zambia. The scale at which any particular variable is measured influences its predictive value, and measuring at a finer scale is not always beneficial. For example, 90 m SRTM elevation data did a superior job at representing potential water flow across the landscape by limiting the influence of factors such as differences in canopy height. Given that the model present here is from a limited study area the generalizability still requires testing on a wider extent. As landscape and climate patterns change from rolling hills and a semi-arid environment to flatter, more sub-humid environment to the north and south, the model will begin to be less successful in its predictions. Current efforts are underway with epidemiologic studies to examine spatial variation in the prevalence of malaria infection relative to predicted mosquito breeding sites in the area to determine if the relationship does exist. In addition, further surveys for predicted breeding sites are being conducted by independent researchers to directly evaluate the predictions.

In 2003, Zambia initiated a new national malaria control initiative Artemether/lumefantrine chemotherapy was selected as the first-line treatment for malaria, and then bed nets and rapid diagnostic testing were introduced [32]. The final phase of the new national malaria control initiative will include residual house spraying. Bed nets have been recently (October 2007) introduced into the Nachiko Study Area, and this is associated with a continued decline of malaria transmission in addition to that seen since the introduction of artemether/lumefantrine anti-malarial chemotherapy. If these measures were to be combined with residual spraying for adult mosquitoes and larviciding, a significant stride in malaria control could be achieved [6,33]. Targeted larviciding along drying river beds when larval sites are limited, may be feasible through landscape analyses and the residual *An. arabiensis *population can be reduced to drastically lower mosquito population come the subsequent rainy season. Models from this study and terrain classifications can be uploaded into newer GPS units with software such as ArcPad (ESRI, Redlands, CA) and be used to navigate to areas with a high potential to contain aquatic habitats where targeted treatments can be implemented.

## Competing interests

The authors declare that they have no competing interests.

## Authors' contributions

JAC was involved in the planning and conducting larval collections, the rearing and identifying mosquitoes, and the statistical analyses and drafted the manuscript. AK participated in larval collection. MM helped rear and identify mosquitoes. CS obtained funding and partook in planning and project oversight. GEG was involved in planning, participated in the interpretation of statistical analyses and took part in editing the manuscript. Each author has read and approved the final manuscript.
